# Caregivers’ burden and schizophrenia patients’ quality of life: Sequential mediating effects of expressed emotion and perceived expressed emotion

**DOI:** 10.3389/fpsyt.2022.961691

**Published:** 2022-08-25

**Authors:** Yicheng Wei, Yanan Peng, Yan Li, Lanjun Song, Kang Ju, Juzhe Xi

**Affiliations:** ^1^Shanghai Key Laboratory of Mental Health and Psychological Crisis Intervention, Affiliated Mental Health Center (ECNU), School of Psychology and Cognitive Science, East China Normal University, Shanghai, China; ^2^Shanghai Changning Mental Health Center, Shanghai, China

**Keywords:** schizophrenia, care burden, quality of life, expressed emotion, family wellbeing

## Abstract

**Background:**

Increasing attention has been paid to the role of caregivers’ burden in affecting quality of life (QoL) of schizophrenic patients. However, less is known about potential mediation mechanisms underlying this relationship. The current study aimed to explore the sequential mediating effect of expressed emotion and perceived expressed emotion on the relationship between care burden and QoL among people with schizophrenia.

**Methods:**

135 Chinese families (one patient and one caregiver) participated in this study. Caregivers reported their care burden and expressed emotion, patients reported their perceived expressed emotion and QoL.

**Results:**

The results of the correlation analysis showed that care burden was negatively related to patients’ QoL, including physical, psychological, and social relationships domains, with patients’ sex, age, educational level, employment status, and medication-taking as covariates. The sequential mediating effects of criticism and perceived criticism between care burden and QoL were not significant. However, the sequential mediating effects of emotional over-involvement and perceived emotional over-involvement (EOI) between care burden and QoL (including physical and psychological domain) were significant.

**Conclusion:**

The results indicated that reducing the burden and expressed emotion of caregivers could be helpful to improve schizophrenia patients’ QoL.

## Introduction

The 2017 Global Burden of Disease report showed that about 20 million people worldwide are affected by schizophrenia (1). According to the National Health Commission (NHC) of the People’s Republic of China (2), the number in China is more than 4 million. Presently, the elimination of symptoms is no longer the only goal of schizophrenia treatment; the social function and adaptation of people with schizophrenia is also an essential issue (3). Quality of life (QoL), which reflects the individual’s perception of their life status, is a good proxy for social adaptation (4).

Family members are caregivers for many outpatients with schizophrenia (5). The caring experience may bring physical, emotional, and social pressure, which results in a psychological state called caregiver burden (6, 7). Specifically, caregivers of people with schizophrenia tend to face more challenges than those of patients with other disorders. They reported higher levels of subjective care burden (8, 9) and experienced poorer mental and physical health (5, 10). Previous research has focused on factors that exacerbate the care burden (both caregiver-level and patient-level factors) and the impact of the care burden on the caregivers (5, 6, 10–13). However, the caregiver burden could also be a predictor of the patient’s recovery, considering that family is a system that affects every member living in it. A few studies have attempted to explore the relationship between caregiver burden and recovery in schizophrenia. Levene et al. (14) found that the care burden reported by caregivers at discharge could positively predict the patient’s symptoms after 9 months. Nuttall et al. (15) found a covariation in change over time in the caregiver burden and QoL. These pieces of evidence suggested that caregiver burden may be one of the essential family factors affecting the QoL of schizophrenia patients.

Care burden may affect patients’ QoL through family interaction, and expressed emotion may play an important role. Expressed emotion refers to the attitudes and emotions of relatives toward mentally ill family members (16–18). It is comprised of three components: emotional over-involvement (EOI), criticism, and hostility. Expressed emotion is a robust predictor of schizophrenia relapse. Specifically, patients who live in a high expressed emotion family had higher rates of relapse. This conclusion has been supported by evidence from many empirical studies and meta-analyses (19–22). A number of studies had also found that care burden was significantly positively correlated with expressed emotion, and caregivers with high expressed emotion reported higher care burden scores (23–27).

As a member of the family system, people with schizophrenia could also feel and perceive the expressed emotion of caregivers during interactions with them. Patients are not simply passive recipients of other peoples’ behavior (28). According to Lazarus’ stress influence model, individuals’ appraisals mediate the relationship between stress and the outcome of stress (29). Therefore, it is also necessary to include patients’ perceptions of expressed emotion in research. On the one hand, previous studies have found that patients’ perceptions of expressed emotion were significantly related to their recovery. For example, caregivers’ criticism reported by first-episode schizophrenia adolescents negatively predicted their QoL (30). Patients who rated their caregivers to be low in care (i.e., indifferent or rejecting) or high in protection (i.e., controlling or intrusive) had more severe symptoms compared with those who reported their caregivers as high in care and low in protection (31). Such a relationship was also found between patient relapse and expressed emotion of institutional caregivers (32). On the other hand, in some comparative studies, patients’ perceived expressed emotion was a better predictor of relapse than relatives’ expressed emotion. Lebell et al. (33) found that patients’ feelings toward their family members and their perceptions of relatives’ attitudes toward them were significantly related to patients’ relapse during a 1-year-follow up, whereas caregiver’s self-reported attitudes did not predict the outcomes. Compared to caregivers’ expressed emotions, patients’ perceptions of criticism and EOI show a more robust predictive effect to relapse in some studies (34–36). In a word, the patients’ perception of expressed emotion should not be overlooked in exploring the relationship between care burden and patients’ QoL.

A meta-analysis revealed that people with schizophrenia in China had a significantly poorer QoL than healthy controls; factors associated with their poorer QoL include diagnostic criteria, study location, female gender, older age, and inpatient status (37). However, these factors focus more on the patient’s situation and lack the family system’s perspective. In China, most people with schizophrenia live with their families (38), and their QoL may also be associated with caregivers. On the other hand, Confucian values require people to be responsible to their families, and the burden of caregivers in Chinese schizophrenia families may be even heavier (39). Caregivers who perceive more burden may show higher levels of expressed emotion (40). Several studies found that expressed emotion was related to relapse in Chinese samples of people (41–43). Given the family system and expectation on caregivers of people with schizophrenia in China, we believe it is essential to explore the relationship between Chinese caregiver burden and patients’ QoL from the perspective of family interaction.

This study aimed to investigate the mechanism between caregiver burden and patients’ QoL by examining the sequential mediating effect of caregiver expressed emotion and patients’ perception of expressed emotion in the Chinese context. In summary, the hypotheses of this study were as follows:

Hypothesis 1: Caregiver burden can negatively predict patients’ QoL.

Hypothesis 2: Caregiver burden can positively predict caregivers’ expressed emotion.

Hypothesis 3: Caregivers’ expressed emotion can positively predict patients’ perception of expressed emotion.

Hypothesis 4: Patients’ perception of expressed emotion can negatively predict patients’ QoL.

Hypothesis 5: Caregivers’ expressed emotion and patients’ perception of expressed emotion play sequential mediating effects between caregiver burden and patients’ QoL.

## Materials and methods

### Procedure and participants

Participants were recruited from Changning district in Shanghai, China. The inclusion criteria of patients were as follows: (1) were 18 years and older; (2) diagnosed with schizophrenia at a local mental health center according to Chinese Classification of Mental Disorder-3 (CCMD-3), which was edited by the Chinese Psychiatric Association according to the clinical description and diagnostic criteria of ICD-10 (44); (3) living with family members; (4) were in good condition and had sufficient capacity to participate in the study according to the evaluation of a qualified psychiatrist. The inclusion criteria of caregivers were as follows: (1) living with the patients; (2) being the primary caregivers of patients.

The research assistant would visit the family of schizophrenics in the company of community doctors (the staff at the mental health center) when the doctors went to the family to do a routine check. The research assistant briefly introduced the study to those patients and their family members after the community doctor finished the visit. If the family was interested in participating in the study, the research assistant would explain the purpose and procedure of the study, data confidentiality and security, participants’ rights, and incentives for the study. If more than one caregiver was interested in participating in the study, we would invite the primary caregiver (who is responsible for taking care of the health of the patient) to fill in the questionnaire. Then the research assistant conducted informed consent and assessment with both participants (patient and caregiver). All participants signed a paper version of the informed consent document. All the questionnaires were administered at a single time point. The family would get a gift valued at 50 *yuan* (the unit of currency in China) as a reward for participation. The study was approved by the authors’ university committee on human research protection as well as the mental health hospital’s ethics committee.

**FIGURE 1:**
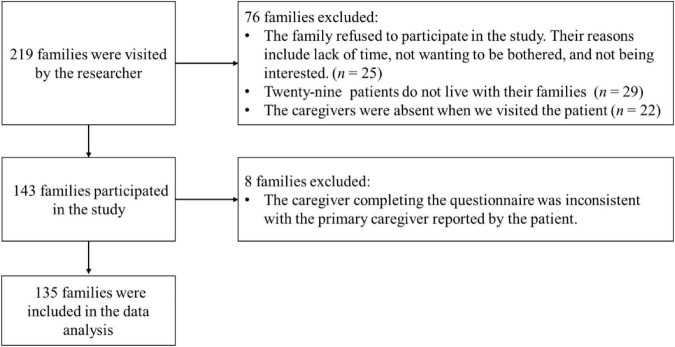
Flowchart of the participants selection.

A total of 219 families were approached by the researcher and eventually 135 families’ data (one patient and one caregiver) were included in the analysis. The process of participant selection is summarized in Figure 1. The demographic information of patients and their caregivers are shown in Table 1.

**TABLE 1:** Demographic information of patients (*N* = 135) and their caregivers (*N* = 135).

Patients	% (*n*)/*M* (*SD*)	Caregivers	% (*n*)/*M* (*SD*)
**Age**	42.78 (8.06)	**Age**	66.28 (11.19)
**Years of illness**	19.42 (8.62)		
**Number of hospitalizations**	1.61 (2.10)		
**Gender**		**Gender**	
Male	50.37% (68)	Male	51.85% (70)
Female	49.63% (67)	Female	48.15% (65)
**Education level**		**Education level**	
Primary school	2.96% (4)	Primary school	13.33% (18)
Junior high school	34.07% (46)	Junior high school	39.26% (53)
High school	41.48% (56)	High school	35.56% (48)
Undergraduate	17.04% (23)	Undergraduate	8.89% (12)
Did not report	4.44% (6)	Did not report	2.96% (4)
**Marital status**		**Caregivers**	
Unmarried	71.11% (96)	Father	40.74% (55)
First marriage	21.48% (29)	Mother	37.78% (51)
Single after divorce	5.93% (8)	Spouse	17.04% (23)
Remarry after divorce	0.74% (1)	Siblings	4.44% (6)
Did not report	0.74% (1)		
**Employment status**		**Monthly per capita household income (*yuan*)**	
Employed	13.33% (18)	Under 3,000	17.78% (24)
Unemployed	85.93% (116)	3,001–5,000	66.67% (90)
Did not report	0.74% (1)	5,001–10,000	11.85% (16)
**Taking psychiatric medication**		More than 10,000	2.22% (3)
Yes	89.63% (121)	Did not report	1.48% (2)
No	9.63% (13)		
Did not report	0.74% (1)		

Monthly per capita household income refers to the average monthly income per person in the family (the patient and the caregivers are living together). It was calculated as the family total income a month divide by the number of people in the family, and this data was reported by the caregiver. yuan is the unit of currency in China. The average monthly income of Shanghai citizens in 2020 is 6,019 yuan.

### Measurement

#### Caregiver burden

Caregiver burden was assessed using the Zarit Burden Interview Chinese version (45). Twenty-two items were rated on a 5-point Likert scale from 0 (*never*) to 4 (*always*), and higher scores indicated a higher level of burden. The Cronbach’s alpha was set at 0.95 in the present study.

#### Expressed emotion

Caregivers’ expressed emotion was measured by the Family Questionnaire (FQ) (46). This questionnaire has shown good psychometric properties in different cultures (47–50). In this study, we translated the questionnaire into Chinese following the back-translation procedure. Translation and back translation procedure was applied to translate the FQ into Chinese. To start, two bilingual psychologists with advanced knowledge of the English language and Chinese as their native language independently translated the questionnaire into Chinese (forward translation). Then, a reconciliation meeting was conducted to develop a consensus version (reconciliated Chinese version) with the help of a third reviewer. After that, two psychologists who were blinded to the original version translated the reconciliated Chinese version back into English (backward translation). A third reviewer compared the backward translation and the original English version and determined there were no significant discrepancies between the two versions. The questionnaire consists of two subscales: EOI (10 items) and criticism (10 items). All items were rated on a 4-point Likert scale from 1 (*strongly disagree*) to 4 (*strongly agree*). The specific items are presented in Supplementary material. Higher scores indicated higher expressed emotion. The Cronbach’s alphas of EOI and criticism were 0.90 and 0.87, respectively. We also performed the confirmatory factor analysis to test the validity of this Chinese version. The model provided a good fit to the data, χ^2^*/df* = 1.63, RMSEA = 0.07, CFI = 0.92, TLI = 0.91, SRMR = 0.07, and the factorial loading of all items was significant (*p* < 0.001).

#### Perceived expressed emotion

We measured patients’ perception of expressed emotion by adapting the FQ. In order to reduce the burden on the patients who participated in the study, we shortened our questionnaire. We selected eight items from the initial twenty items of the FQ based on factor loading while taking into consideration language variety. Specifically, we analyzed the factor loading results of the two subscales in Wiedemann et al. (46). For the subscale for EOI, we selected items based on the rank of factor loadings, with the highest factor loading item selected first. If one item has a similar meaning to the last item in the ranking of factor loadings, we would skip this item and chose another item. For example, the item “He/she irritates me.” (With the second highest loadings among the items of criticism) was literally similar to the item “I’m often angry with him/her” (the item with top loading factor). Thus “He/she irritates me” was not chosen. The item selection procedure for the criticism subscale followed a similar procedure. After that, we adapted the items into a patient report version. For example, the item “I’m often angry with him/her” was adapted into “He/she is often angry with me.” The specific items are presented in Supplementary material. These eight items were also rated on a 4-point Likert scale from 1 (*strongly disagree*) to 4 (*strongly agree*). Higher scores indicated a higher perceived level of expressed emotion. The Cronbach’s alphas of perceived EOI and perceived criticism were both 0.83. The result of confirmatory factor analysis showed that the model provided an acceptable fit to the data, χ^2^*/df* = 2.98, RMSEA = 0.12, CFI = 0.92, TLI = 0.88, SRMR = 0.06, and the factorial loading of all items was significant (*p* < 0.001).

#### Quality of life

QoL was measured using three subscales of the World Health Organization Quality of Life (WHOQOL-BREF) questionnaire Chinese version (51). This measurement has four subscales: physical domain, psychological domain, social relationships, and environmental domain. We selected items from the physical, psychological, and social relationship domains, to reduce the response burden for patients (52). The environmental domain was excluded because it is more affected by the public environment than the family system. We also removed an item (Are you satisfied with your sex life?) that measured social relationships. This item is difficult to answer for the patients because most people with schizophrenia are unmarried or single. In the current study, the Cronbach’s alphas of the physical, psychological, and social relationship domain were 0.77, 0.78, and 0.86.

### Data analysis

Data were analyzed using SPSS 23.0 and Mplus 7.0. The code for Mplus is presented in Supplementary material. The rate of missing data for the demographic information ranged from 0.74 to 4.44% among patients and from 1.48 to 4.44% among caregivers. The rate of missing data was 1.48% for care burden and 0.74% for the social relationship domain of QoL. There were no missing data for the other main study variables. Full information maximum likelihood (FIML) was used to handle missing data in the analyses. Analyses were performed in three steps. First, we calculated the mean and standard deviation of all variables and the Pearson correlation between variables. Second, we examined the direct effect of caregiver burden on patients’ QoL. Third, we examined the sequential mediating effects of caregivers’ expressed emotion and patients’ perceptions of expressed emotion between burden and patients’ QoL.

## Results

Demographic information is presented in Table 1. The average age of the patients was 42.78 ± 8.06 years, 50.37% were male, the average years of illness was 19.42 ± 8.62 years and number of hospitalizations was 1.61 ± 2.10. The average age of the caregivers was 66.28 ± 11.19 years, 51.85% were male and 78.52% were the parents of the patients.

Table 2 displays the means and standardized deviation of all variables and the Pearson relation between all variables. All correlations are statistically significant. We used Mplus to analyze the sequential mediating effects of caregivers’ expressed emotion and patients’ perceptions of expressed emotion. All variables and outcomes were standardized. First, we examined the prediction of caregivers’ expressed emotion to patients’ QoL with patients’ sex, age, educational level, employment status, and medication-taking as covariates (these covariates were significantly related to patients’ QoL, the estimates for coefficients of covariates are presented in Supplementary material). Supporting hypothesis 1, we found that caregiver burden could significantly negatively predict QoL of patients, including: physical domain [β = −0.39, *p* < 0.001, 95% CI = (−0.543, −0.239)], psychological domain [β = −0.35, *p* < 0.001, 95% CI = (−0.506, −0.190)], and social relationships domain [β = −0.18, *p* = 0.046, 95% CI = (−0.357, −0.004)].

**TABLE 2:** Descriptive statistics and correlations (*N* = 135).

Variables	*M*	*SD*	1	2	3	4	5	6	7
1 Caregivers’ burden	1.44	0.72	1						
2 Caregivers’ EOI	2.63	0.42	0.61**	1					
3 Caregivers’ criticism	2.44	0.47	0.62**	0.70**	1				
4 Patients’ perceived EOI	2.72	0.54	0.39**	0.61**	0.50**	1			
5 Patients’ perceived criticism	2.29	0.53	0.38**	0.38**	0.65**	0.52**	1		
6 Patients’ QoL (physical domain)	3.39	0.47	−0.44**	−0.34**	−0.41**	−0.36**	−0.29**	1	
7 Patients’ QoL (psychological domain)	3.22	0.50	−0.41**	−0.45**	−0.41**	−0.41**	−0.34**	0.73**	1
8 Patients’ QoL (social relationships)	3.28	0.66	−0.26**	−0.35**	−0.34**	−0.25**	−0.22**	0.70**	0.73**

**p < 0.01.

**FIGURE 2:**
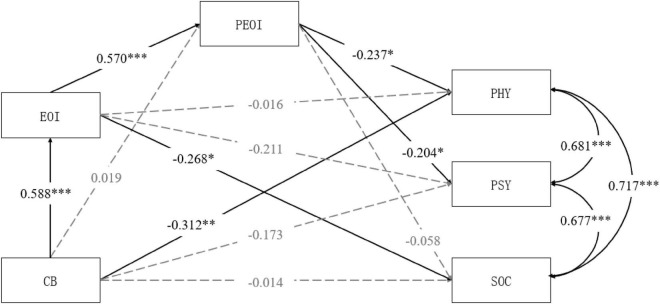
The mediating model of EOI and PEOI between CB and QoL (PHY, PSY, and SOC). CB, care burden; EOI, caregivers’ emotional over-involvement; PEOI, patients’ perceived emotional over-involvement; PSY, psychological domain of patients’ QoL; SOC, social relationships domain of patients’ QoL. *p < 0.05, ^**^p < 0.01, ^***^p < 0.001.

Second, we examined the sequential mediating effect of caregivers’ EOI and patients’ perception of EOI. We fitted a model where care burden served as the independent variable, caregivers’ EOI and patients’ perception of EOI as mediators, and the three subscales of QoL as dependent variables. The patients’ sex, age, educational level, employment status, and medication-taking were control variables. The results are shown in Figure 2. The model provided a good fit to the data, χ^2^*/df* = 1.35, RMSEA = 0.05, CFI = 0.99, TLI = 0.96, SRMR = 0.04. Caregiver burden significantly positively predicted caregivers’ EOI, supporting hypothesis 2. Caregivers’ EOI significantly positively predicted patients’ perception of EOI, supporting hypothesis 3. Patients’ perception of EOI significantly negatively predicted the psychical and psychological domain of QoL. However, the path between patients’ perception of EOI and social relationships was not significant. Hypothesis 4 was only partially supported. We also tested the significant levels of indirect effects in the model. As shown in Table 3, the sequential mediating effects of caregivers’ EOI and patients’ perception of EOI on the relationship between care burden and patients’ physical and psychological QoL were significant. However, the sequential mediating effect on the relationship between care burden and social relationships domain was not significant. Therefore, hypothesis 5 was only partially supported.

**TABLE 3:** Standardized mediating path analysis.

Path	β	*SE*	95%CI	*p*
**CB→PHY**	−**0.312**	**0.094**	**[**−**0.497,** −**0.128]**	**0.007**
CB→EOI→PHY	–0.009	0.054	[−0.135, 0.116]	0.883
CB→PEOI→PHY	–0.004	0.022	[−0.047, 0.038]	0.837
**CB→EOI→EOI→PHY**	−**0.080**	**0.035**	**[**−**0.148,** −**0.011]**	**0.023**
CB→PSY	–0.173	0.096	[−0.361, 0.016]	0.073
CB→EOI→PSY	–0.124	0.065	[−0.252, 0.004]	0.058
CB→PEOI→PSY	–0.004	0.019	[−0.040, 0.033]	0.838
**CB→EOI→PEOI→PSY**	−**0.068**	**0.034**	**[**−**0.136,** −**0.001]**	**0.046**
CB→SOC	–0.014	0.107	[−0.223, 0.195]	0.897
**CB→EOI→SOC**	−**0.158**	**0.072**	**[**−**0.299,** −**0.017]**	**0.029**
CB→PEOI→SOC	–0.001	0.006	[−0.012, 0.010]	0.847
CB→EOI→PEOI→SOC	–0.020	0.035	[−0.089, 0.050]	0.579
**CB→PHY**	−**0.260**	**0.097**	**[**−**0.450,** −**0.071]**	**0.007**
CB→CC→PHY	–0.124	0.070	[−0.262, 0.013]	0.077
CB→PCC→PHY	0.001	0.004	[−0.007, 0.009]	0.815
CB→CC→PCC→PHY	–0.012	0.038	[−0.087, 0.063]	0.755
**CB→PSY**	−**0.201**	**0.100**	**[**−**0.396,** −**0.006]**	**0.043**
CB→CC→PSY	–0.121	0.071	[−0.261, 0.019]	0.090
CB→PCC→PSY	0.003	0.009	[−0.014, 0.020]	0.742
CB→CC→PCC→PSY	–0.035	0.040	[−0.112, 0.043]	0.382
CB→SOC	–0.005	0.108	[−0.217, 0.206]	0.961
**CB→CC→SOC**	−**0.183**	**0.077**	**[**−**0.334,** −**0.032]**	**0.018**
CB→PCC→SOC	0.000	0.004	[−0.007, 0.007]	0.928
CB→CC→PCC→SOC	0.004	0.042	[−0.078, 0.086]	0.926

Control variables: patients’ sex, age, educational level, employment status, and medication-taking. CB, care burden; EOI, caregivers’ emotional over-involvement; PEOI, patients’ perceived emotional over-involvement; CC, caregivers’ criticism; PCC, patients’ perceived criticism; PHY, physical domain of patients’ QoL; PSY, psychological domain of patients’ QoL; SOC, social relationships domain of patients’ QoL. Bolded values in a row mean that a path is statistically significant.

**FIGURE 3:**
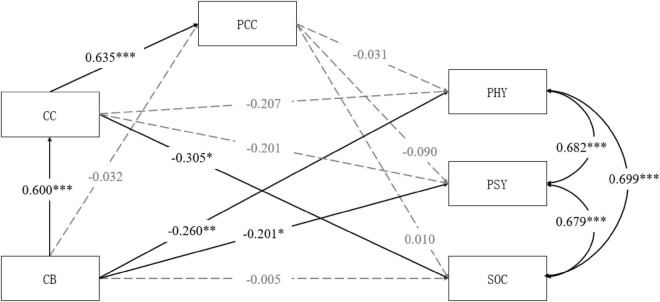
The mediating model of CC and PCC between CB and QoL (PHY, PSY, and SOC). CB, care burden; CC, caregivers’ criticism; PCC, patients’ perceived criticism; PHY, physical domain of patients’ QoL; PSY, psychological domain of patients’ QoL; SOC, social relationships domain of patients’ QoL. *p < 0.05, ^**^p < 0.01, ^***^p < 0.001.

Third, we examined the sequential mediating effect of caregivers’ criticism and patients’ perception of criticism. We fitted a model where care burden served as the independent variable, caregivers’ criticism and patients’ perception of criticism were mediators, and the three subscales of QoL were dependent variables. The patients’ sex, age, educational level, employment status, and medication-taking were control variables. The results are shown in Figure 3. The model provided a good fit to the data, χ^2^*/df* = 0.94, RMSEA = 0.00, CFI = 1.00, TLI = 1.00, SRMR = 0.03. Caregiver burden significantly positively predicted caregivers’ criticism, supporting hypothesis 2. Caregivers’ criticism significantly positively predicted patients’ perception of criticism, supporting hypothesis 3. Patients’ perception of criticism did not significantly predict the three domains of QoL. The direct and indirect effects of all paths are shown in Table 3. The results indicated that the sequential mediating effects of caregivers’ criticism and patients’ perception of criticism on the relationship between care burden and patients’ QoL were non-significant.

## Discussion

The current study aimed to explore the relationship between care burden and the QoL of people with schizophrenia and the family interaction process. Specifically, we examined the sequential mediating effects of caregivers’ expressed emotion (EOI and criticism) and patients’ perception of expressed emotion (perceived EOI and perceived criticism) on the relationship between care burden and patients’ QoL (physiological domain, psychological domain, and social relationships domain). Hypothesis 1 was supported by our results. Care burden significantly negatively predicted the physical, psychological, and social relationships domain of patients’ QoL. Hypothesis 2 was supported. Care burden significantly positively predicted the EOI and criticism of caregivers. Hypothesis 3 was supported. The EOI and criticism of caregivers significantly positively predicted patients’ perceptions of EOI and criticism. Hypothesis 4 was partially supported. The patients perceived emotional involvement significantly negatively predicted their physical and psychological QoL, but not the social relationships domain. The predictive effect of patients’ perceived criticism on QoL was non-significant. Hypothesis 5 was partially supported. The sequential mediating effects of caregivers’ EOI and patients’ perception of EOI on the relationship between care burden and patients’ physical and psychological QoL were significant. However, the sequential mediating effects of caregivers’ criticism and patients’ perception of criticism were non-significant.

The regression analysis results showed that the care burden significantly negatively predicted the patients’ QoL. It was consistent with the results of previous studies (15, 53). A study in Chinese families of people with schizophrenia also found that higher caregiving burden was associated with poorer family functioning (54), which means it would be hard for caregivers to give a patient enough social support and care. Caregivers may also be unable to support the patient to go out and return to society, which leads to a poorer QoL for patients. This was also found in an empirical study with a Chinese sample (55).

The path analysis of the mediating model showed that care burden significantly positively predicted expressed emotion and expressed emotion also positively predicted patients’ perceptions of expressed emotion. Previous studies have found that caregivers who reported high care burden also show a high level of expressed emotion (23–26). This is consistent with the cross-sectional data results in our study. A longitudinally designed study also found that change in burden is associated with the change in EE among relatives of people with schizophrenia (56). The direction of effect in the association of burden and EE could not be determined from our study or existing studies. A possible explanation is that caregivers of individuals with schizophrenia often feel overwhelmed, stressed, drained, burdened, frustrated, or angry (5). A meta-analysis showed that feelings and emotions are at the core of the caregiver’s caring experience (57). Under these circumstances, coping skills to help manage pressure and negative feelings would be important for caregivers to improve their interaction with the patient. However, those who reported high subjective care burden may lack coping skills brought on by the caring role (58). They may therefore experience worry and frustration with the patient and then express these emotions to the patient.

Although some studies indicated that schizophrenia patients have difficulties in recognizing others’ emotions (59, 60), research on expressed emotion showed that people with schizophrenia can still detect and perceive emotions and attitudes of close family members (24, 61). Therefore, it is reasonable that expressed emotion of family members positively predicted patients’ perceived expressed emotion in this study.

The results of our study found that EOI and patients’ perceived EOI served as sequential mediators on the relationship between care burden and patients’ QoL. However, the other aspect of expressed emotion, criticism, did not show such effects. This difference might result from patients’ different appraisals toward caregivers’ EOI and criticism in Chinese culture. Expressed emotion is related to relapse of schizophrenia because high expressed emotion in the family environment is a kind of stress for patients (21). In our study, nearly 80% of the caregivers were parents. Asian parents tend to show more control and critical attitudes when they raise their children; they also tend to express their dissatisfaction with children due to high expectations for them (62, 63). People with schizophrenia in China may get used to parents’ criticism and not appraise it as pressure. Thus, in our study, patients’ perception of criticism did not significantly predict their QoL. However, the mediating effects of EOI and patients’ perceived EOI were significant. The impact of EOI may be due to its impairment on patients’ agency. Caregivers high in EOI tend to perceive the patient as less capable of participating and completing tasks associated with the recovery process (64). This may lead the patients to lose confidence in getting back to society and reduce participation in activities that help improve their QoL. Some studies found that expressed emotion did not significantly predict schizophrenia patients’ relapse when caregivers’ perception of patients’ agency was controlled (65). Cross-cultural studies of expressed emotion also found differences in the predictive effects of EOI and criticism on schizophrenia relapse (66, 67).

### Strengths and limitations

This study also had several limitations. First, it was not possible to infer causality due to the cross-sectional nature of the study. Longitudinal design could be more helpful to explore the causal relationship between care burden and QoL of people with schizophrenia. Second, the sample in our study consisted of patients with different ages and years of illness duration. The average age was 43. The finding from the current study may not translate to other samples, such as first-episode schizophrenia or early-phase schizophrenia. Third, although we have controlled for multiple covariates in the mediation analysis, unmeasured confounders may also affect the mediation effects of expressed emotion and perceived expressed emotion. For example, some individual traits related to coping, such as resilience, might confound patients’ perceived EE and QoL. The illness belief about schizophrenia (whether it could be controlled or not) might be a confounder of caregivers’ burden and expressed emotion. Future studies could explore the mediating effects of EE and patients’ perceived EE by measuring and controlling known confounders.

Despite limitations in this study, our study has several strengths. To our knowledge, the current study is the first to explore the relationship between care burden and patients’ QoL by examining the sequential mediating effects of expressed emotion and perceived expressed emotion. By including the measure of patients’ perceptions of expressed emotion, the current study provided insight into the family interaction’s impact on the QoL of schizophrenia patients. The present study also implied that the two aspects of expressed emotion (i.e., EOI and criticism) might have different effects on patients’ QoL, which expands our understanding of expressed emotion and enriches relevant empirical evidence. Future studies can further explore whether schizophrenia patients’ appraisal of EOI and criticism is different in a Chinese cultural context. In addition, whether different roles of caregivers may moderate the impact of care burden on patients’ QoL *via* expressed emotion could also be explored in future studies, given that a caregiving experience of a parent might be different from that of a spouse. The results of our study also provide some implications for improving the QoL of patients with schizophrenia. Psychoeducation targeted for caregivers could be helpful to reduce their level of expressed emotion, which may enhance patients’ QoL. Additional support systems and resources (such as social workers or professional caregivers) may aid Chinese families in reducing care burden and improve QoL for patients with schizophrenia.

## Conclusion

In summary, our results examined the sequential mediating effects of expressed emotion and patients’ perception of expressed emotion on the relationship between care burden and the QoL of people with schizophrenia. Care burden was negatively related to patients’ QoL, including the physical, psychological, and social relationships domain. The EOI of caregivers and patients’ perception of EOI served as mediators between care burden and patients’ QoL. However, criticism of caregivers and patients’ perception of criticism did not mediate this effect.

## Data availability statement

The raw data supporting the conclusions of this article will be made available by the authors, without undue reservation.

## Ethics statement

The studies involving human participants were reviewed and approved by the East China Normal University. The patients/participants provided their written informed consent to participate in this study.

## Author contributions

YW and YP analyzed and interpreted the data and drafted the manuscript. YL, LS, and KJ contacted participants and collected data. JX designed the study and revised the manuscript. All authors have read and approved the final manuscript.
