# Supplementary material for: Caregivers’ burden and schizophrenia patients’ quality of life: Sequential mediating effects of expressed emotion and perceived expressed emotion
Source: Front Psychiatry. 2022 Aug 25;13:961691. doi: 10.3389/fpsyt.2022.961691 (PMC9454947; doi:10.3389/fpsyt.2022.961691)
Supplement: Supplementary file 1 [file Data_Sheet_1.PDF]

Supplementary Material

1 Supplementary codes

The Mplus codes for mediating model

1 The sequential mediating effect of caregivers' EOI and patients' perception of EOI

```
  DATA: FILE IS data for mplus 3.0.dat;
  VARIABLE:
            MISSING ARE ALL (-999);
NAMES ARE index_p P_sex P_age P_education P_marital P_employe
P_hospitalization_P_yearofill eoi_p cc_p physi_p psych_p soci_p
index_r R_sex R_age R_education R_role R_income R_medication
    eoi_r cc_r zbi_r;

     USEVARIABLE = zbi_r eoi_r eoi_p physi_p psych_p soci_p
P_sex P_age P_education P_employed R_medication;
MODEL:
physi_p on zbi_r eoi_r eoi_p;
        psych_p on zbi_r eoi_r eoi_p;
        soci_p on zbi_r eoi_r eoi_p;
        eoi_r on zbi_r;
        eoi_p on eoi_r zbi_r;

        physi_p with psych_p;
        physi_p with soci_p;
        psych_p with soci_p;
```

```
    physi_p on P_sex P_age P_education P_employed R_medication;
    psych_p on P_sex P_age P_education P_employed R_medication;
    soci_p on P_sex P_age P_education P_employed R_medication;

  MODEL INDIRECT:
    physi_p ind zbi_r;
    psych_p ind zbi_r;
    soci_p ind zbi_r;

  output: SAMPSTAT STDYX CINTERVAL;
```

2 The sequential mediating effect of caregivers' criticism and patients' perception of criticism

```
DATA:
  FILE IS data for mplus 3.0.dat;
VARIABLE:
  MISSING ARE ALL (-999);
  NAMES ARE index_p P_sex P_age P_education P_marital P_employe
P_hospitalization_P_yearofill eoi_p cc_p physi_p psych_p soci_p
index_r R_sex R_age R_education R_role R_income R_medication
   eoi_r cc_r zbi_r;

  USEVARIABLE = zbi_r cc_r cc_p physi_p psych_p soci_p
  P_sex P_age P_education P_employed R_medication;
```

```
MODEL:physi_p on zbi_r cc_r cc_p;
    psych_p on zbi_r cc_r cc_p;
    soci_p on zbi_r cc_r cc_p;
    cc_r on zbi_r;
    cc_p on cc_r zbi_r;
    physi_p with psych_p;
    physi_p with soci_p;
    psych_p with soci_p;

    physi_p on P_sex P_age P_education P_employed R_medication;
    psych_p on P_sex P_age P_education P_employed R_medication;
    soci_p on P_sex P_age P_education P_employed R_medication;

MODEL INDIRECT:
    physi_p ind zbi_r;
    psych_p ind zbi_r;
    soci_p ind zbi_r;

output:SAMPSTAT STDYX CINTERVAL;
```

2 Supplementary Figures and Tables

2.1 Supplementary Figures

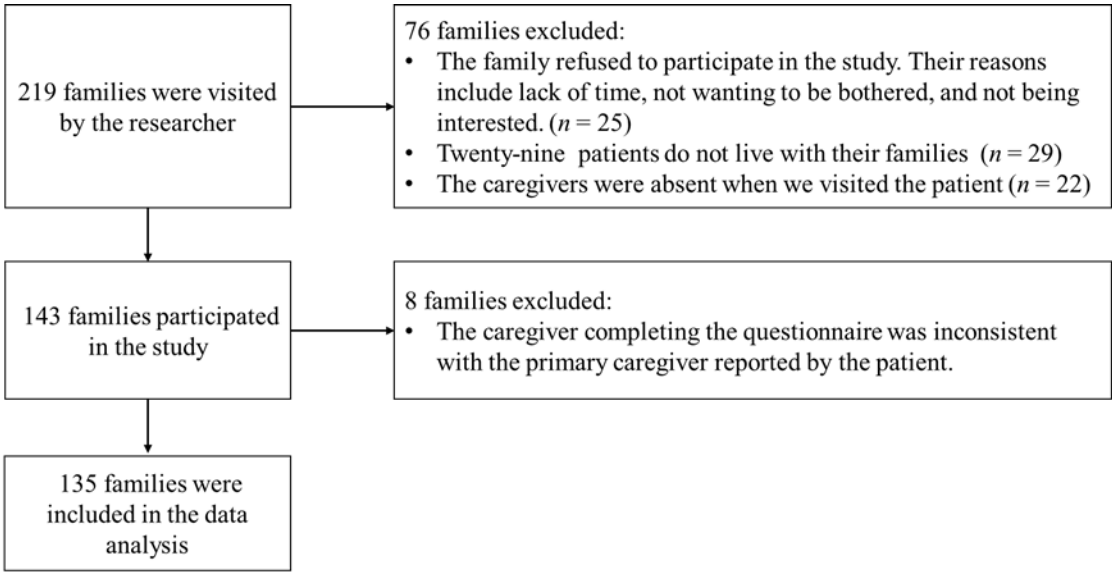

Supplementary Figure 1. Flowchart of the participants selection

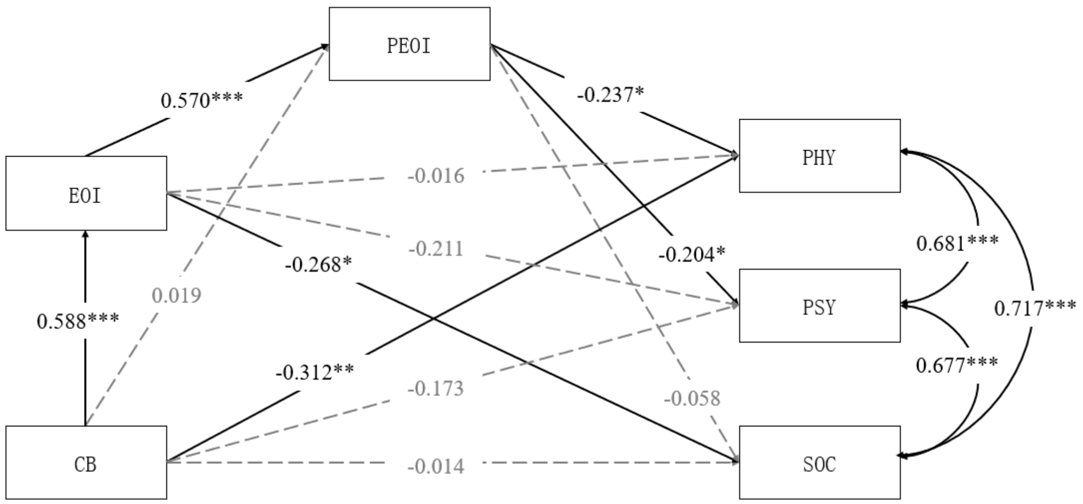

Supplementary Figure 2. The mediating model of EOI and PEOI between CB and QoL (PHY, PSY, and SOC).

Note: CB = care burden; EOI = caregivers' emotional over-involvement; PEOI = patients' perceived emotional over-involvement; PSY = psychological domain of patients' QoL; SOC = social relationships domain of patients' QoL.

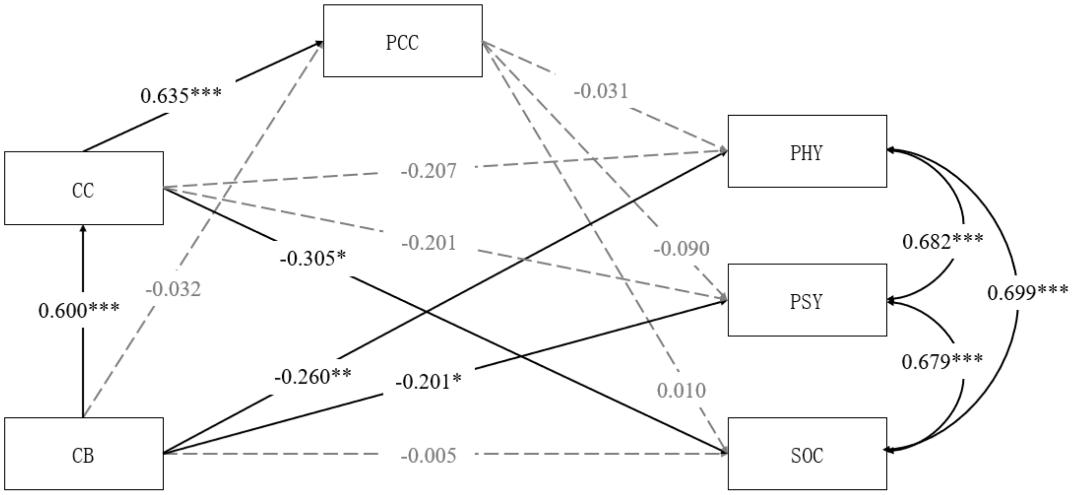

Supplementary Figure 3. The mediating model of CC and PCC between CB and QoL (PHY, PSY, and SOC).

Note: CB = care burden; CC = caregivers’ criticism; PCC = patients’ perceived criticism; PHY = physical domain of patients’ QoL; PSY = psychological domain of patients’ QoL; SOC = social relationships domain of patients’ QoL.

2.2 Supplemental Table 1

Items measuring expressed emotion and perceived expressed emotion

Items for caregiver	Items for patient
1.I tend to neglect myself because of him/her	1.He/she often thinks about what is to become of me.
2.I have to keep asking him/her to do things	2.It's hard for us to agree on things.
3.I often think about what is to become of him/her	3.He/she regard his/her own needs as less important.
4.He/she irritates me	4.He/she feels that I sometimes get on his/her nerves.
5.I keep thinking about the reasons for his/her illness	5.He/she is very worried about me.
6.I have to try not to criticize him/her	6.He/she always ask me to behave differently.
7.I can't sleep because of him/her	7.He/she has given up important things in order to be able to help me.
8.It's hard for us to agree on things	8.He/she is often angry with me.
9.When something about him/her bothers me, I keep it to myself	
10.He/she does not appreciate what I do for him/her	
11.I regard my own needs as less important	
12.He/she sometimes gets on my nerves	
13.I'm very worried about him/her	
14.He/she does some things out of spite	
15.I thought I would become ill myself	
16.When he/she constantly wants something from me, it annoys me	
17.He/she is an important part of my life	
18.I have to insist that he/she behave differently	
19.I have given up important things in order to be able to help him/her	
20.I'm often angry with him/her	

Note: The items with odd number are items for emotional over-involvement/perceived emotional over-involvement, and items with even number are items measuring criticism/perceived criticism.

Supplemental Table 2

The estimates for coefficients of covariates in the mediating model of EOI

Path	*β*	*SE*	95%CI	*p*
SEX→PHY	0.090	0.076	[-0.059, 0.240]	0.238
**AGE→PHY**	**-0.216**	**0.078**	**[-0.370, -0.063]**	**0.006**
EDUCATION→PHY	0.154	0.080	[-0.003, 0.311]	0.054
EMPLOYED→PHY	-0.066	0.079	[-0.220, 0.089]	0.404
MEDICATION→PHY	0.136	0.078	[-0.018, 0.290]	0.083
SEX→PSY	-0.015	0.077	[-0.167, 0.136]	0.844
AGE→PSY	-0.079	0.080	[-0.236, 0.077]	0.321
EDUCATION→PSY	0.123	0.081	[-0.035, 0.281]	0.128
EMPLOYED→PSY	-0.131	0.079	[-0.286, 0.024]	0.097
MEDICATION→PSY	-0.053	0.080	[-0.208, 0.103]	0.509
SEX→SOC	0.048	0.085	[-0.118, 0.214]	0.572
AGE→SOC	-0.048	0.088	[-0.221, 0.125]	0.587
EDUCATION→SOC	0.095	0.089	[-0.080, 0.271]	0.286
EMPLOYED→SOC	-0.162	0.086	[-0.331, 0.007]	0.060
MEDICATION→SOC	0.010	0.087	[-0.162, 0.181]	0.911

Note: Covariates are patients' sex, age, educational level, employment status, and medication-taking. EOI = emotional over-involvement; PHY = physical domain of patients' QoL; PSY = psychological domain of patients' QoL; SOC = social relationships domain of patients' QoL.

Supplemental Table 3

The estimates for coefficients of covariates in the mediating model of CC

Path	*β*	*SE*	95%CI	*p*
SEX→PHY	0.076	0.077	[-0.075, 0.226]	0.325
AGE→PHY	-0.187	0.079	[-0.343, -0.032]	0.018
EDUCATION→PHY	0.189	0.079	[0.035, 0.344]	0.016
EMPLOYED→PHY	-0.080	0.079	[-0.235, 0.076]	0.316
MEDICATION→PHY	0.114	0.082	[-0.046, 0.274]	0.163
SEX→PSY	-0.051	0.078	[-0.204, 0.103]	0.516
AGE→PSY	-0.034	0.082	[-0.194, 0.127]	0.681
EDUCATION→PSY	0.171	0.081	[0.013, 0.328]	0.034
EMPLOYED→PSY	-0.139	0.081	[-0.297, 0.019]	0.084
MEDICATION→PSY	-0.062	0.083	[-0.225, 0.102]	0.458
SEX→SOC	0.017	0.084	[-0.148, 0.181]	0.843
AGE→SOC	-0.011	0.088	[-0.183, 0.160]	0.897
EDUCATION→SOC	0.127	0.087	[-0.044, 0.299]	0.144
EMPLOYED→SOC	-0.162	0.086	[-0.331, 0.006]	0.059
MEDICATION→SOC	-0.010	0.089	[-0.185, 0.165]	0.913

Note: Covariates are patients' sex, age, educational level, employment status, and medication-taking. CC = criticism; PHY = physical domain of patients' QoL; PSY = psychological domain of patients' QoL; SOC = social relationships domain of patients' QoL.
